# An Integrated Method for Coding Trees, Measuring Tree Diameter, and Estimating Tree Positions

**DOI:** 10.3390/s20010144

**Published:** 2019-12-24

**Authors:** Linhao Sun, Luming Fang, Yuhui Weng, Siqing Zheng

**Affiliations:** 1 Key Laboratory of Forestry Intelligent Monitoring and Information Technology Research of Zhejiang Province, Zhejiang A & F University, Lin’an 311300, Zhejiang, China; Acesunlh@126.com (L.S.);; 2 School of Information Engineering, Zhejiang A & F University, Lin’an 311300, Zhejiang, China; 3 Arthur Temple College of Forestry and Agriculture, Stephen F. Austin State University, Nacogdoches, TX 75962, USA; wengy@sfasu.edu

**Keywords:** forest inventory, quick response code technique, ultra-wide band technology, angle sensor

## Abstract

Accurately measuring tree diameter at breast height (DBH) and estimating tree positions in a sample plot are important in tree mensuration. The main aims of this paper include (1) developing a new, integrated device that can identify trees using the quick response (QR) code technique to record tree identifications, measure DBH, and estimate tree positions concurrently; (2) designing an innovative algorithm to measure DBH using only two angle sensors, which is simple and can reduce the impact of eccentric stems on DBH measures; and (3) designing an algorithm to estimate the position of the tree by combining ultra-wide band (UWB) technology and altitude sensors, which is based on the received signal strength indication (RSSI) algorithm and quadrilateral localization algorithm. This novel device was applied to measure ten 10 × 10 m square plots of diversified environments and various tree species to test its accuracy. Before measuring a plot, a coded sticker was fixed at a height of 1.3 m on each individual tree stem, and four UWB module anchors were set up at the four corners of the plot. All individual trees’ DBHs and positions within the plot were then measured. Tree DBH, measured using a tree caliper, and the values of tree positions, measured using tape, angle ruler, and inclinometer, were used as the respective reference values for comparison. Across the plots, the decode rate of QR codes was 100%, with an average response time less than two seconds. The DBH values had a bias of 1.89 mm (1.88% in relative terms) and a root mean square error (RMSE) of 5.38 mm (4.53% in relative terms). The tree positions were accurately estimated; the biases on the x-axis and the y-axis of the tree position were −8.55–14.88 cm and −12.07–24.49 cm, respectively, and the corresponding RMSEs were 12.94–33.96 cm and 17.78–28.43 cm. The average error between the estimated and reference distances was 30.06 cm, with a standard deviation of 13.53 cm. The device is cheap and friendly to use in addition to its high accuracy. Although further studies are needed, our method provides a great alternative to conventional tools for improving the efficiency and accuracy of tree mensuration.

## 1. Introduction

Diameter at breast height (DBH), measured at a height of 1.3 m on the bole of a tree, is the most commonly measured tree attribute [1,2,3], whether for inventory, management, or research purposes, since many tree and forest attributes [4,5], such as basal area, volume [6], and stand density [7], are derived from DBH measurements [8]. Therefore, developing tools that can measure tree DBH accurately, efficiently, and conveniently is always desirable.

Foresters rely on a variety of conventional dendrometers, such as diameter tapes (D-tapes) and tree calipers, to measure tree DBH. These tools are often based on the geometry of circles; trees are presumed to have circular cross-sections [9], although this is rarely true in reality [10,11]. Most tree stems violate this assumption to some level, either in the form of an oval, an ellipse, or a closed convex [12,13,14,15], resulting in biases (mostly overestimates) in DBH when measured by diameter tapers or tree calipers (which often take one measurement) [16,17]. D-tapes are more commonly used for measuring permanent sample plots because they are perceived as being more consistent for repeated DBH measurements [18], while calipers are often preferred for DBH measurements in temporary plots [19]. In tree mensuration, to reduce the impact of eccentric cross-section of a tree, it is often recommended to measure the major and minor diameters of the tree and obtain the average diameter of the two measurements as the tree DBH, which, in literature, is often used as the reference data for comparison purpose [20]. There are other limits to using a D-tape or caliper to measure tree DBH, such as the manual recording of measurements [16,17], thereby reducing efficiency and increasing mistakes in measurement and data entry [18,19]. Both tools, while relatively reliable, are labor-intensive. Some advanced, electronic measurement devices for DBH measurement [20,21,22,23,24], such as electronic tree-measuring forks and electronic tapes, have been developed. The electronic tapes do not solve the problem of eccentric cross-section, while electronic tree-measuring forks are based on an ultrasonic system [25,26], which can be easily affected by environments [27,28,29]. More recently, some methods for measuring DBH using non-contact methods, including terrestrial laser scanning (TLS) [30,31,32,33,34], mobile phones with time-of-flight (TOF) cameras [4,35,36], and close-range photogrammetry (CRP) [1,22,37], have been proposed. These methods use point clouds to extract data, which are expensive [28] and require high computational capacity of microprocessors, limiting their application in forestry inventory. Other than the method of CRP, estimating tree DBH based on the TLS and TOF methods is still based on the assumption that trees are circular in cross section.

Other than tree size measurements, identifying trees and estimating the positions of the trees in a sample plot are important elements for measuring sampling plots [27,28], and this is particularly true for permanent inventory plots where successive measurements are always needed. Conventionally, foresters either use markers or tags to identify trees. This method, while still widely used, is labor-intensive. The spatial information, i.e., the tree positions, represents important information regarding the stand density, as well as in studies of distance-dependent growth and the dynamics of trees and stands. While tree position information is difficult to obtain, studies have shown that the individual tree positions can be determined using a total station or ultrasonic trilateration technology [25,26]. New developments in integrating DBH measurement and tree position estimation, using non-contact methods such as TLS [30,31,32,33,34], TOF cameras [4,35,36], and CRP [1,22,37], have been reported. However, results of these methods can easily be affected by the stand environments such as stand density, abundance of shrubs and vegetation, and light conditions. They are also time-consuming, labor-intensive, and require complicated procedures in data processing [38]. Therefore, many practical and technical restrictions still exist in applying these methods in forestry inventories. 

There is a pressing need to develop an integrated system that can identify trees, measure tree DBH, and estimate tree positions concurrently, accurately, and efficiently in terms of both labor and cost. The recent developments in computer science and electronics make this possible. Quick response (QR) code technology [39] is now widely used due to its characteristics of a high capacity in encoding data, strong damage resistance, and fast decoding [39,40,41,42]. The ultra-wide band (UWB) technology is based on sending and receiving carrier-less radio impulses using extremely accurate timing, and it is particularly suitable for estimating distance and positions [43,44]. This technology has constantly gained interest thanks to its high accuracy (i.e., a typical error of 30 cm or less), making it more attractive than other wireless technologies, such as WiFi, ZigBee, and Bluetooth, which can normally estimate the location with an accuracy of several meters [45,46,47,48]. In this paper, we integrated these technologies into a device to identify trees, measure tree DBH, and estimate tree positions concurrently. The device was applied to measure trees in forest plots of different environments and tree species to evaluate its accuracy and efficiency.

## 2. Technology and Theory

### 2.1. Design of the Main Device 

**Figure 1:**
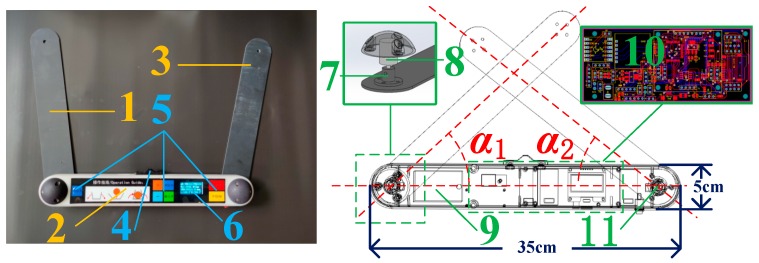
The main device and its components: 1, the left arm; 2, the middle beam; 3, the right arm; 4, the vertex; 5, the keys; 6, the display panel; 7, the flange; 8, the first angle sensor; 9, the battery; 10, the printed circuit board (PCB); and 11, the second angle sensor.

The main device and its components can be found in Figure 1, and their attribute descriptions are listed in Table 1. The device consists of a microprocessor, an analog-to-digital sampling (ADS) module, a QR scanner, a secure digital memory card (SD card), an interface, Bluetooth, a power management circuit, a keyboard interface, an altitude sensor, a display interface, and a UWB module. Note that the angle between the left arm and the middle beam is defined as α_1_ and that between the middle beam and right arm as α_2_ (Figure 1). The geometric centers of the vertex and middle component are on the same line.

### 2.2. Technology of Coding Trees

**Figure 2:**
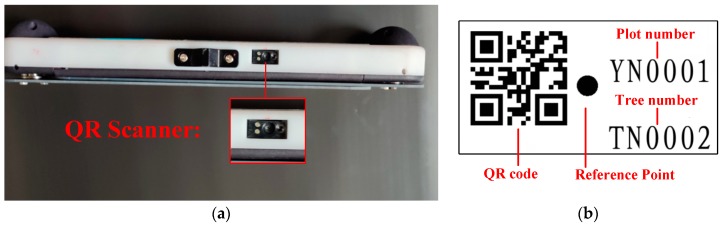
The QR scanner and an example of a tree code: (**a**) QR scanner (**b**) coded sticker

A QR scanner (Figure 2a) is used to decode the QR code (encoded by NiceLabel 2017 Barcode software [19]), which is printed on a sticker (Figure 2b). Other information, such as the identification (ID) numbers of the plot and the tree and the reference point, can also be found on the sticker. For example, for the QR code of “TN0002 | YN0001” (Figure 2b); “TN0002” is the tree ID, and “YN0001” is the sample plot ID. Note that, if stickers are not used, the tree-codes will be generated automatically in the process of measuring trees’ DBH and positions.

### 2.3. Angle Calculation

As shown in Figure 1, tree DBH is measured by measuring α_i_ (i = 1, 2) values. When the central axis of the angle sensor rotates, the change in the angle has a linear relationship with the change in the output voltage signal. Therefore, the voltage signals from ADS modules can be converted to α_i_ (i = 1, 2) using the following equation:

(1) $$\alpha_{i}=\frac{360^{\circ }}{\mathrm{Un}}\times (Uc_{i}-Vz_{i}\frac{\mathrm{Un}}{\mathrm{Vr}}),$$

where U_n_ is the ADS value of the current input voltage of the two angle sensors, U_ci_ is the ADS value of the output voltage of the i^th^ angle sensor, V_zi_ is the reference value of the output voltage of the i^th^ angle sensor at initialization when α_i_ equals 0°, and V_r_ is the reference value of the input voltage of the two angle sensors at initialization.

### 2.4. Double-Sided Two-Way Ranging

**Figure 3:**
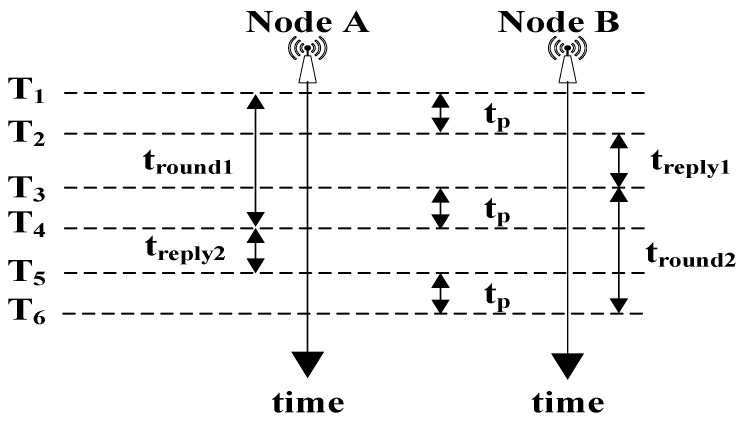
Scheme of double-sided, two-way ranging (DS-TWR).

The basic ranging principle of the UWB modules used in this method is double-sided two-way ranging (DS-TWR) [46,47,48,49,50]. Clock drift correction and signal power error may affect the accuracy of the position. The DS-TWR is an additional round of communication based on single-sided two-way ranging (SS-TWR) [46,47,48,49,50,51]. The time of the two types of communication can compensate each other for the errors caused by the clock offset [46,47,48,49,50,51,52] and signal power error [51,52] to improve the accuracy of the DS-TWR. Figure 3 presents an example of calculating the distance (Dis) between two nodes (A and B). The distance between node A and node B can be calculated as follows:

(2) $$\mathrm{Dis}\;=c\times t_{p}=c\times \frac{t_{round1}\times t_{round2}-t_{reply1}\times t_{reply2}}{t_{round1}+t_{round2}+t_{reply1}+t_{reply2}},$$

where node A and node B are two communication UWB nodes; Dis is the distance between node A and node B; t_p_ is the time of the wireless signal propagation in the air; c is the speed of light in the air; time is the time axis; T_1_ is the time when node A sends the first pulse; T_2_ is the time when node B receives the first pulse; T_3_ is the time when node B sends the first pulse; T_4_ is the time when node A receives the first pulse; T_5_ is the time when node A sends the second pulse; T_6_ is the time when node B receives the second pulse; t_round1_ is the total time of node A sending and receiving pulses in the first round of communication; t_reply1_ is the reply time for node B in the first round of communication; t_round2_ is the total time of node B sending and receiving pulses in the second round of communication; t_reply2_ is the reply time for node A in the second round of communication.

## 3. Materials and Methods 

### 3.1. Study Area

This study was carried out in Lin’an (N30°15′, E119°43′), China. We selected 10 square plots with a size of 10 × 10 m. These plots varied greatly in average tree DBH, environment, and tree species composition (Table 2). Plots 1–5 were located at the Botanical Garden of Zhejiang A&F University, dominated by artificial forest and turf. Plots 6–10 were located in the suburb of Lin’an, dominated by natural forest, dense weeds, and shrubs.

### 3.2. Methods

#### 3.2.1. The System Workflow

**Figure 4:**
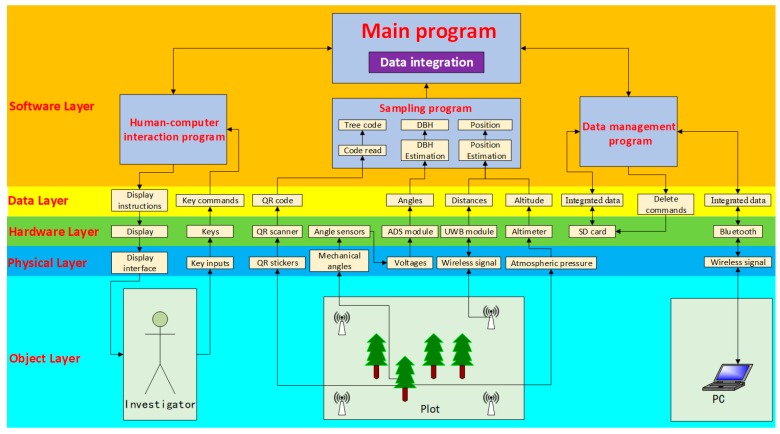
The system workflow of the device for coding trees and estimating diameter.

As shown in Figure 4, the system flow, from top to bottom, consisted of the software layer, data layer, hardware layer, physical layer, and object layer. A few subprograms, including a human–computer interaction program, which is used for keyboard input and display control, a sampling program, which is used for reading tree codes and DBH values and tree position data extraction, and a data management program, which is used for encoding data storage and data communication, were incorporated into the software layer. After completing an individual tree’s measurement (see Section 3.2.2), the tree ID, its DBH value, and position were automatically saved to the system.

#### 3.2.2. The Operation Workflow 

**Figure 5:**
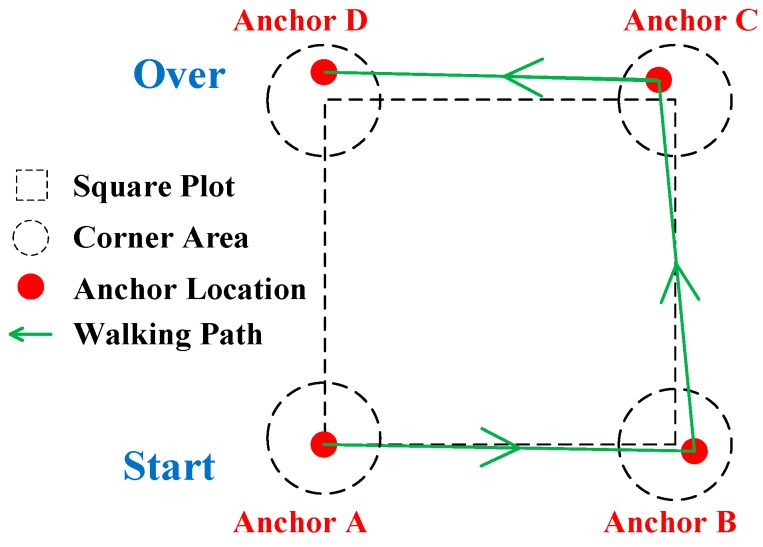
Anchors set up in a square plot.

**Figure 6:**
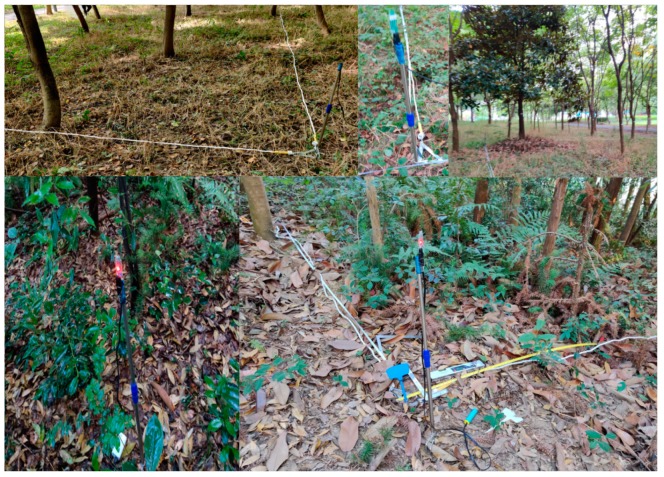
An example to show the anchor installation in a plot.

To complete the measurement of a plot, the following tools and instruments are needed: the device, coded stickers, a nail gun, short nails, and four UWB modules (referred to as anchors A, B, C, and D). The coded stickers were fixed at a height of 1.3 m on individual trees in the plot. The anchors (Figure 5 and Figure 6) were set up at plot corners as follows: first, anchor A was set up in one corner of the plot, and then the device was used to measure the altitude of corner A (H_A_). Second, anchor B was set up in a neighboring plot corner of A, and the altitude of the corner was measured (H_B_) as well as the distance between anchors A and B (Dis_AB_). The same step was repeated to set up anchors C and D in the remaining plot corners, and their altitudes (H_C_ and H_D_) were measured as well as the distances between corners (Dis_AC_, Dis_BC_, Dis_AD_, Dis_BD_, and Dis_CD_).

**Figure 7:**
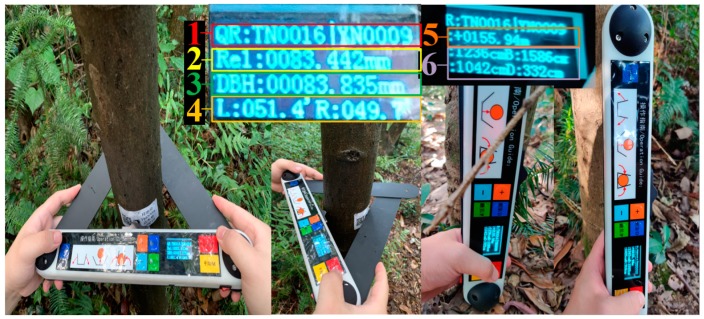
Measurement of a tree. On the display screen, line 1 represents the tree code; line 2, the diameter at breast height (DBH) value of the previously measured tree; line 3, the measured DBH value; line 4, the values of α_1_ and α_2_; line 5, the value of H_n_; and line 6, the values of A_n_, B_n_, C_n_, and D_n_.

A tree was measured as follows (Figure 7): first, the investigator scanned the tree QR code, followed by measurement of the tree DBH twice at two different directions (mainly followed the major and minor DBH directions). For each measure, values of α_1_ and α_2_ were automatically used to calculate the tree DBH (see Section 3.2.3). The average of the two measurements was used as the tree DBH. Finally, the device was placed at the bottom of the tree to obtain H_n_, A_n_, B_n_, C_n_, and D_n_. Here, H_n_ is the altitude of the device, A_n_, B_n_, C_n_, or D_n_ is the distance between anchors A, B, C, or D and the device, respectively, and n refers to the nth tree in the plot. The above steps were repeated to finish measuring all the trees in the plot. Similar to the DBH value, each tree’s position was estimated twice, and the average was used as the tree position value. The measurement data were then uploaded to a person computer (PC) for further analysis.

#### 3.2.3. Measurement Algorithm of a Tree’s DBH

**Figure 8:**
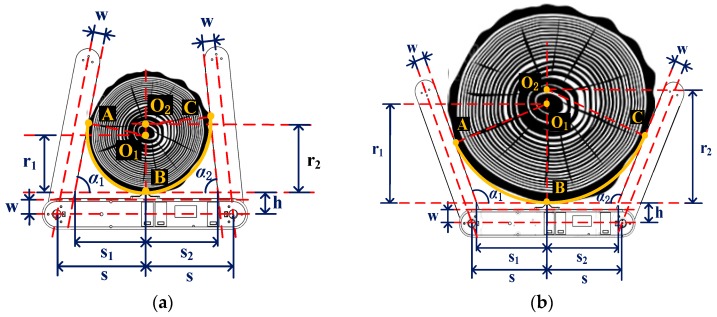
Measurement method of tree DBH: (**a**) for a small tree and (**b**) a large tree.

Figure 8 provides an example of measuring tree DBH using the device, with A, B, and C being the contact points of the device’s arms and beam against the tree trunk, which results in two arcs, arc BA and arc BC. Since the cross-section surface may be eccentric, arcs BA and BC have different centers, O_1_ and O_2_. Note that, for the device, the values of w (2.5 cm), h (3.5 cm), and s (15 cm) are fixed mechanical structural values (Figure 8). 

Here, w equals the half width of the arms or beam; h equals w plus the maximum width of the vertex; and s equals the half distance between the axes of two angle sensors.

In order to obtain the value of the tree DBH, the s_i_ (i = 1,2) values are needed, which can be calculated using Taylor’s expansion in the following equations:

(3) $$s_{i}\;=\{\begin{array}{ll} s-\frac{h\times (1-\frac{{\theta_{i}}^{2}}{2!}+\frac{{\theta_{i}}^{4}}{4!}-\frac{{\theta_{i}}^{6}}{6!})+w}{\theta_{i}-\frac{{\theta_{i}}^{3}}{3!}+\frac{{\theta_{i}}^{5}}{5!}-\frac{{\theta_{i}}^{7}}{7!}} & \left(\alpha_{i}\neq 90^{\circ }\right)\\ s-w & \left(\alpha_{i}=90^{\circ }\right) \end{array}.$$

where θ_i_ = α_i_ × 3.141592/180 and I = 1, 2.

The calculated s_i_ can then be used to calculate the radius, r_i_, using Taylor’s expansion, in the following equations:

(4) $$r_{i}\;=\{\begin{array}{ll} s_{i}\times \frac{\frac{\theta_{i}}{2}-\frac{{\theta_{i}}^{3}}{2^{3}\times 3!}+\frac{{\theta_{i}}^{5}}{2^{5}\times 5!}-\frac{{\theta_{i}}^{7}}{2^{7}\times 7!}}{1-\frac{{\theta_{i}}^{2}}{2^{2}\times 2!}+\frac{{\theta_{i}}^{4}}{2^{4}\times 4!}-\frac{{\theta_{i}}^{6}}{2^{6}\times 6!}} & \left(\alpha_{i}\neq 90^{\circ }\right)\\ s-w & \left(\alpha_{i}=90^{\circ }\right) \end{array}.$$

The tree DBH is then calculated as the total of r_1_ and r_2_:

(5) $$\mathrm{DBH}=r_{1}+r_{2}.$$

Since r_1_ and r_2_ are calculated based on different arcs, the impacts of eccentric tree cross-sections on measuring DBH would be accounted for, resulting in a more accurate estimate than that obtained using conventional dendrometers.

#### 3.2.4. Estimation Algorithm of a Tree Position

**Figure 9:**
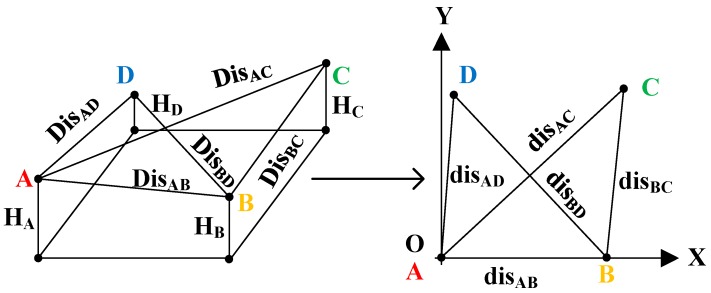
Conversion from a three-dimensional coordinate to a two-dimensional coordinate in tree position estimation.

In order to obtain a tree’s position in a plot, the spatial coordinate system of four anchors is first transformed into a plane rectangular coordinate system OXY (Figure 9). 

The plane OXY is perpendicular to the gravity direction, the origin O is the location of anchor A, and the x-axis is the projection direction from anchor A to anchor B. The three-dimensional scalars H_A_, H_B_, H_C_, H_D_, Dis_AB_, Dis_BC_, Dis_AC_, Dis_AD_, and Dis_BD_ are then transformed into two-dimensional scalars in the OXY plane.

(6) $$\begin{array}{l} {\mathrm{dis}}_{\mathrm{AB}}=\sqrt{{{\mathrm{Dis}}_{\mathrm{AB}}}^{2}{-(H_{A}-H_{B})}^{2}}\\ {\mathrm{dis}}_{\mathrm{BC}}=\sqrt{{{\mathrm{Dis}}_{\mathrm{BC}}}^{2}{-(H_{B}-H_{C})}^{2}}\\ {\mathrm{dis}}_{\mathrm{AC}}=\sqrt{{{\mathrm{Dis}}_{\mathrm{AC}}}^{2}{-(H_{A}-H_{C})}^{2}}\\ {\mathrm{dis}}_{\mathrm{AD}}=\sqrt{{{\mathrm{Dis}}_{\mathrm{AD}}}^{2}{-(H_{A}-H_{D})}^{2}}\\ {\mathrm{dis}}_{\mathrm{BD}}=\sqrt{{{\mathrm{Dis}}_{\mathrm{BD}}}^{2}{-(H_{B}-H_{D})}^{2}} \end{array}$$

The coordinates of anchors A, B, C, and D can then be obtained.

(7) $$\begin{array}{l} \left(X_{A},Y_{A})=(0,0\right)\\ \left(X_{B},Y_{B})=(0,{\mathrm{dis}}_{\mathrm{AB}}\right)\\ \left(X_{C},Y_{C})=(\frac{{{\mathrm{dis}}_{\mathrm{AB}}}^{2}+{{\mathrm{dis}}_{\mathrm{AC}}}^{2}-{{\mathrm{dis}}_{\mathrm{BC}}}^{2}}{2{\mathrm{dis}}_{\mathrm{AB}}},\sqrt{{{\mathrm{dis}}_{\mathrm{AC}}}^{2}-{\left(\frac{{{\mathrm{dis}}_{\mathrm{AB}}}^{2}+{{\mathrm{dis}}_{\mathrm{AC}}}^{2}-{{\mathrm{dis}}_{\mathrm{BC}}}^{2}}{2{\mathrm{dis}}_{\mathrm{AB}}}\right)}^{2}}\right)\\ \left(X_{D},Y_{D})=(\frac{{{\mathrm{dis}}_{\mathrm{AB}}}^{2}+{{\mathrm{dis}}_{\mathrm{AD}}}^{2}-{{\mathrm{dis}}_{\mathrm{BD}}}^{2}}{2{\mathrm{dis}}_{\mathrm{AB}}},\sqrt{{\mathrm{disAD}}^{2}-{\left(\frac{{{\mathrm{dis}}_{\mathrm{AB}}}^{2}+{{\mathrm{dis}}_{\mathrm{AD}}}^{2}-{{\mathrm{dis}}_{\mathrm{BD}}}^{2}}{2{\mathrm{dis}}_{\mathrm{AB}}}\right)}^{2}}\right) \end{array}$$

Similarly, the projection distances a_n_, b_n_, c_n_, and d_n_ can be calculated as follows:

(8) $$\begin{array}{l} a_{n}=\sqrt{{A_{n}}^{2}{-(H_{A}-H_{n})}^{2}}\\ b_{n}=\sqrt{{B_{n}}^{2}{-(H_{B}-H_{n})}^{2}}\\ c_{n}=\sqrt{{C_{n}}^{2}{-(H_{C}-H_{n})}^{2}}\\ d_{n}=\sqrt{{D_{n}}^{2}{-(H_{D}-H_{n})}^{2}} \end{array}$$

Here, a_n_, b_n_, c_n_, and d_n_ represent the projection distances between anchors A, B, C, and D, respectively, and the device in the OXY plane. n refers to the nth tree in the plot.

**Figure 10:**
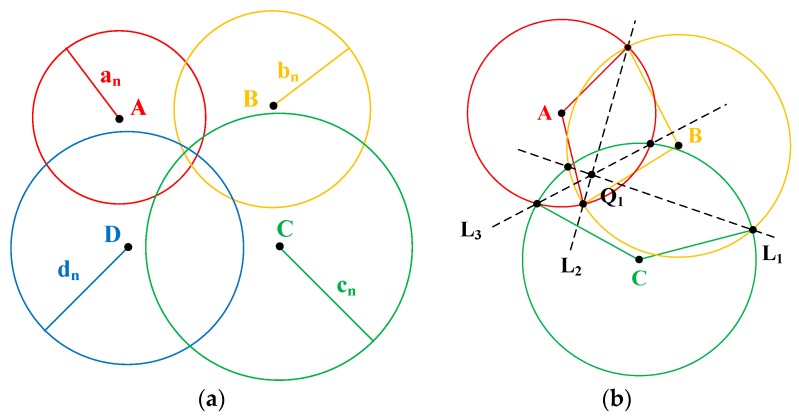
Position estimation using (**a**) quadrilateral localization and (**b**) trilateration localization.

Although UWB wireless signals have very strong penetration ability, in reality, the random ranging error may be caused by humans and/or the tree body [48,49,50,51,52,53], resulting in a prolonged communication time between the UWB nodes. Therefore, the values of a_n_, b_n_, c_n_, and d_n_ may be slightly higher than the respective actual values, and consequently, the four circles may not intersect at one common point (Figure 10a). The received signal strength indication (RSSI) algorithm [50,51,52,53,54,55,56], such as quadrilateral localization (Figure 10a), provides an alternative to solve this problem and to improve the accuracy and precision.

From Figure 10a, we can develop four trilateration localizations with each of three circles (ABC, ABD, ACD, and BCD). Taking ABC (Figure 10b) as an example, where three circles, A, B, and C are intersected, three lines, L_1_, L_2,_ and L_3_, are formed by linking the intersections of any two circles. According to the trilateration localization algorithm [53,54,55,56,57,58], the following equations can be developed:

(9) $$\{\begin{array}{l} 2(X_{A}-X_{B})X_{1}+2(Y_{A}-Y_{B})Y_{1}={b_{n}}^{2}-{a_{n}}^{2}+{X_{A}}^{2}-{X_{B}}^{2}+{Y_{A}}^{2}-{Y_{B}}^{2}\\ 2(X_{B}-X_{C})X_{1}+2(Y_{B}-Y_{C})Y_{1}={c_{n}}^{2}-{b_{n}}^{2}+{X_{B}}^{2}-{X_{C}}^{2}+{Y_{B}}^{2}-{Y_{C}}^{2}\\ 2(X_{A}-X_{C})X_{1}+2(Y_{A}-Y_{C})Y_{1}={c_{n}}^{2}-{a_{n}}^{2}+{X_{A}}^{2}-{X_{C}}^{2}+{Y_{A}}^{2}-{Y_{C}}^{2} \end{array}.$$

The coordinates of Q_1_ (X_1_, Y_1_) can be obtained from Equation (9). By the same token, the coordinates of the three other connecting points of the three other trilateration localizations can be obtained—Q_2_ (X_2_,Y_2_), Q_3_ (X_3_,Y_3_), and Q_4_ (X_4_,Y_4_). Then, according to the RSSI algorithm and quadrilateral localization algorithm [59], the above equations can be used to calculate the coordinates of X_n_ and Y_n_ as follows:

(10) $$\{\begin{array}{c} X_{n}=\frac{\frac{X_{1}}{a_{n}+b_{n}+c_{n}}+\frac{X_{2}}{a_{n}+b_{n}+d_{n}}+\frac{X_{3}}{a_{n}+c_{n}+d_{n}}+\frac{X_{4}}{b_{n}+c_{n}+d_{n}}}{\frac{1}{a_{n}+b_{n}+c_{n}}+\frac{1}{a_{n}+b_{n}+d_{n}}+\frac{1}{a_{n}+c_{n}+d_{n}}+\frac{1}{b_{n}+c_{n}+d_{n}}}\\ Y_{n}=\frac{\frac{Y1}{a_{n}+b_{n}+c_{n}}+\frac{Y2}{a_{n}+b_{n}+d_{n}}+\frac{Y3}{a_{n}+c_{n}+d_{n}}+\frac{Y4}{b_{n}+c_{n}+d_{n}}}{\frac{1}{a_{n}+b_{n}+c_{n}}+\frac{1}{a_{n}+b_{n}+d_{n}}+\frac{1}{a_{n}+c_{n}+d_{n}}+\frac{1}{b_{n}+c_{n}+d_{n}}} \end{array}.$$

#### 3.2.5. Evaluation of the Accuracy of the DBH and Tree Position

The major and minor DBHs of each tree were measured using a caliper in the 10 plots, and their average was used as the reference DBH value for comparison. The accuracy of using the device to measure tree DBH was evaluated by comparison with the reference DBH values and by calculating the error, bias, relative bias, root mean square error (RMSE), relative RMSE, and mean absolute percent error (MAPE), as defined in the following equations:

(11) $$\mathrm{error}\;=d_{j}\;-\;d_{jr}\;,$$

(12) $$\mathrm{BIAS}\;=\frac{\sum_{j=1}^{n}(\;d_{j}\;-\;d_{jr}\;)}{n},$$

(13) $$\mathrm{relBIAS}\;=\frac{\sum_{j=1}^{n}(\;d_{j}\;/d_{jr}-1\;)}{n}\times 100\%,$$

(14) $$\mathrm{RMSE}\;=\sqrt{\frac{\sum_{j=1}^{n}{\left(\;d_{j}\;-d_{jr}\right)}^{2}}{n}},$$

(15) $$\mathrm{relRMSE}\;=\sqrt{\frac{\sum_{j=1}^{n}{(\;d_{j}\;/d_{jr}-1\;)}^{2}}{n}}\times 100\%,$$

(16) $$\mathrm{MAPE}\;=\frac{\sum_{j=1}^{n}\left|\;(d_{j}\;/d_{jr}-1\;)\right|}{n}\times 100\%,$$

where d_j_ is the jth tree DBH measured using the device, d_jr_ is the jth tree reference DBH measured using the caliper, and n is the number of measured trees.

The reference value of the tree position was also measured and converted into the OXY plane coordinate system. First, the plane coordinate of the tree bottom position nearest to the x-axis or y-axis was determined by the measured values of a tape scale, an angle ruler, and an inclinometer. Then, half of the tree DBH value was added to or subtracted from the reference position value of the plane coordinate in the y-axis or x-axis direction. The BIAS and RMSE were calculated to reflect the accuracy of the tree position in the x-axis and y-axis directions, respectively. The errors of the distance (Ed) between the estimated and the reference position values were calculated as follows:

(17) $$\mathrm{Ed}\;=\sqrt{{\left(\;X_{j}-X_{jr}\;\right)}^{2}+{(\;Y_{j}-Y_{jr}\;)}^{2}}.$$

Here, x_j_ and y_j_ are the jth tree position estimators in the x- and y-axis directions, respectively; x_jr_ and y_jr_ are the jth position reference values in the x- and y-axis directions, respectively, of the OXY plane coordinate system.

## 4. Results

### 4.1. Tree Identification

The decoding rate of QR codes from the coded stickers on trees was 100%, and the decoding response time was short, less than two seconds. 

### 4.2. Evaluation of DBH

**Figure 11:**
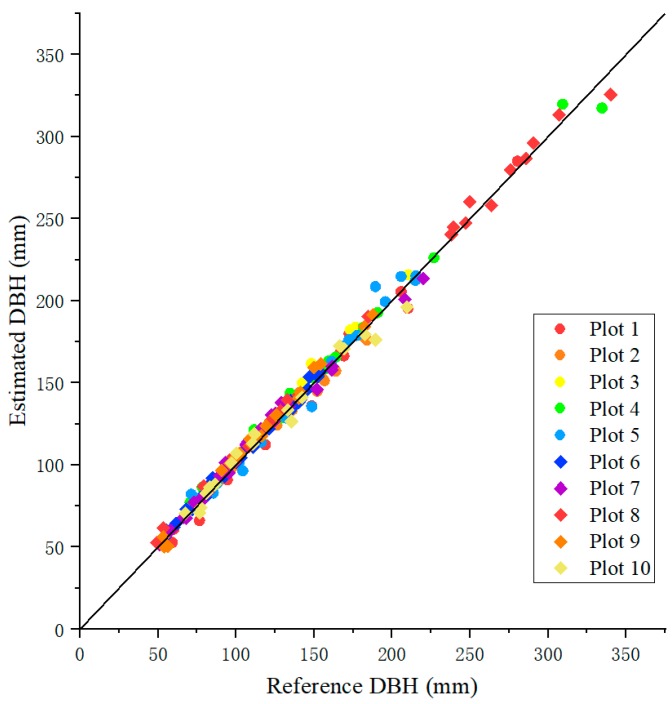
Scatter plot between the measured DBH values using the device and the reference DBHs measured with a caliper.

**Figure 12:**
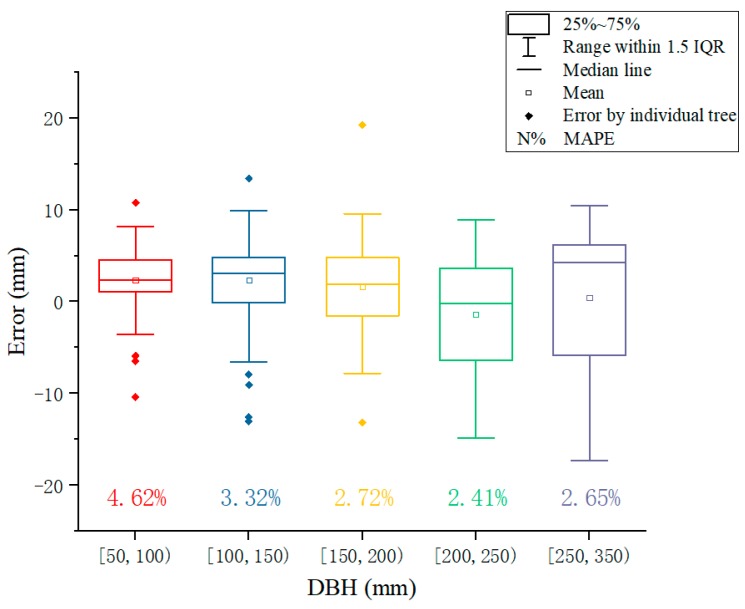
Distribution of the error in DBH for different tree (DBH) sizes.

The DBHs measured by the device were similar to those measured by the caliper (Figure 11), resulting in an overall BIAS of 1.89 mm (1.88%) and an RMSE of 5.38 mm (4.53%) across all plots (Table 3). The BIAS for an individual plot varied from −0.62 (Plot 10) to 5.13 mm (Plot 3), and on a relative term from −0.25 to 3.54%. The RMSE values by plot were small, ranging from 3.48 to 6.84 mm, and from 2.29 to 5.76% in a relative term. Variation in BIAS or RMSE was not strongly related to tree DBH size, tree species, and plot slope (Table 3). Also, the BIAS or RMSE between the plots in the botanical garden (plots 1–5) and those in the natural stands (plots 6–10) were comparable. The error for each tree size distributed normally (Figure 12). There was a trend, however, that as the DBH increased, more variation in error was observed. The smaller DBH groups tended to have larger MAPEs. For the largest DBH group (from 250 to 350 mm), the average error was larger than zero, suggesting that the device overestimated the tree DBH compared to the caliper. 

### 4.3. Evaluation of Tree Position

**Figure 13:**
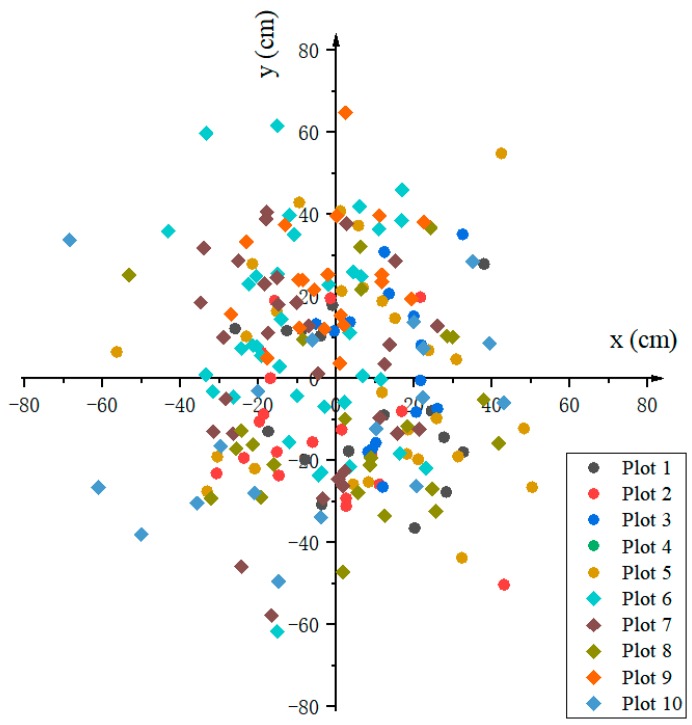
Errors in tree positions measured by the device.

Figure 13 presents the errors of the distance (Ed) between the estimated and corresponding reference position values by individual tree, ranging from 0 to 77 cm in the plane OXY. The bias ranged from approximately −8.55 to 14.88 cm on the x-axis and from −12.07 cm to 24.69 cm on the y-axis (Table 4). The RMSEs in the x-axis (21.85 cm) and y-axis (24.53 cm) directions were similar (Table 4). No significant correlation (approximately −0.26 to 0.30) was found between the errors in the x-axis and y-axis directions. The mean value of Ed was 30.06 cm, with a standard deviation of 13.53 cm across plots and ranged from 23.44 to 38.08 cm by plot (Table 5). The average Ed of plots 1 to 5 was relatively lower than that of plots 6 to 10. Additionally, if the plot had a larger slope or more trees, Ed was relatively larger. Across all plots, the correlation coefficients of the mean Ed were 0.78 with the slope and 0.24 with the stand density. Relatively, slope had relatively more influence on mean Ed than the stand density.

## 5. Discussion

In this study, we reported a device that can identify trees using QR code technology, measure tree DBHs using angle sensors, and estimate tree positions using UWB modules and altitude sensors. Use of the QR code is very common in modern daily life, due to its low cost and advanced technology, but it is rarely used in forestry, especially in inventory plot surveys. To our knowledge, this is the first study to integrate QR code technology into a tree dendrometer to identify trees. In forestry inventories, tree identification data are of high value for retrospective data and database management development.

Conventional dendrometers are only accurate for trees that are circular in cross-section. When a tree cross-section is eccentric, it is recommended to measure the major and minor diameters or two diameters perpendicular to each other, using a tree caliper, and to use their average as the tree DBH value [26]. Different from conventional tools, the device described here takes into account the fact that the cross-sections of trees are not always circular by calculating the radii of different arcs. We evaluated the accuracy of the device in measuring DBH by comparing it to the corresponding DBH values measured twice using a tree caliper. The resulting BIAS and RMSE were small (Table 3), suggesting the device is accurate in measuring tree DBH. Modern tools to measure DBH have been developed to improve accuracy. Liang et al. [8] used a multi-single-scan TLS method to estimate tree DBH. Those authors mapped five dense forest plots, compared the results with manual field measurements, and reported an RMSE range from 0.90 to 1.90 cm. Reference [4] estimated the tree DBH of nine square plots using simultaneous localization and mapping (SLAM) algorithms paired with a time-of-flight (TOF) camera. The DBH estimations had a 0.33 mm (1.78%) BIAS and a 1.26 cm (6.39%) RMSE. Reference [1] used unmanned aerial system-based photogrammetry and terrestrial photogrammetry to estimate tree DBH. The error of the diameter estimation was observed to be less than 1 cm in terms of RMSE. Reference [27] used 3D point clouds of individual trees collected using the CRP method to estimate the tree DBH of four species. The relative RMSE varied from 0.90% to 1.85%, strongly depending on tree species. While the accuracies of the above methods are high and time-efficient, their practical applications in forest inventories, particular in large and dense forests, are still limited since these methods involve complicated procedures in data processing and require specific tools. Even though our method is based on a simple trigonometric theorem, the obtained BIAS (0.19 cm and 1.88%) and RMSE (0.54 cm and 4.53%) (Table 3) were either comparable to or even lower than those reported above. The DBH measurement accuracy of the methods based on TLS, CRP, or TOF may be affected by environmental conditions such as light intensity and stand density. The effects of environmental conditions on our device’s accuracy may be small, which explains why the BIAS and RMSE (Table 3) did not vary substantially with plot slope, tree species, or stand type. 

**Figure 14:**
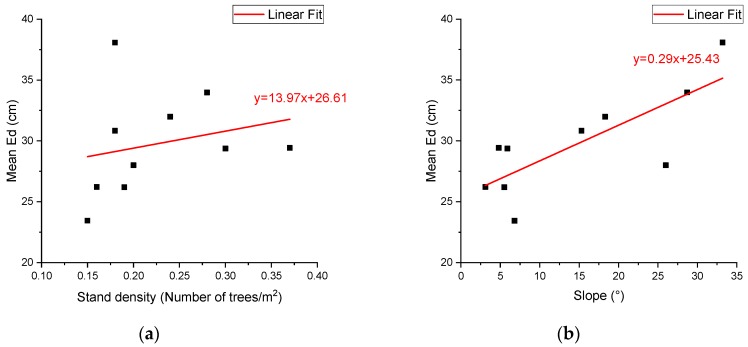
Scatter plot (**a**) between mean Ed and stand density (**b**) and slope.

Our results also suggest that the device can estimate tree positions accurately, with the resulting BIAS (−8.55 to 14.88 cm on the x-axis and −12.07 to 24.49 cm on the y-axis), RMSE (12.94–33.96 cm and 17.78–28.43 cm on the x-axis and the y-axis, respectively), and Ed (30.06 cm) being small. Reference [4] estimated the tree position using the SLAM algorithms paired with a TOF camera and reported an RMSE of 0.12 m, regardless of the axis directions. Their results, however, were based on sample plots on a flat site with no weeds and small shrubs; therefore, the actual use of their method in dense forests needs further verification. While application of SLAM algorithms paired with a TOF seems promising in terms of precision, it is known to be affected by stand environments, such as stand density, abundance of shrubs and vegetation, etc., and involves complicated data processing procedures, limiting its application to forest inventory. Reference [37] reported a position error of less than 0.5 m, although with a systematic shift, and they used five cameras to realize their method, which is impractical for forest surveys. Reference [44] used an extended Kalman filter (EKF) method that integrated inertial navigation system (INS) and UWB data. They tested their method in an indoor area of 10 × 10 m and found that the positioning error of UWB was between 10 and 30 cm and INS-UWB was less than 15 cm. Clearly, our device can obtain similar or more accurate position estimates than the above cited methods, even our device only uses the trilateration localization algorithm, RSSI algorithm, and quadrilateral localization algorithm, which have a much lower complexity in both time and space. Note that the accuracy in estimating tree positions using our device may be affected by stand environments. The slope correlated more positively with Ed than the stand density did (Table 2 and Figure 14). Therefore, further studies are needed to improve accuracy in estimating positions in various environments and find how to reduce influence from slope.

Note that the plot size adopted in this study is 100 m^2^, much smaller than the regular plot size often used in forestry inventory. This raises a concern of using the device to estimate positions in more realistic scenarios since the performance of four UWB sensors would probably degrade when the plot area is large. However, the range accuracy deteriorates only when the distance between two UWB nodes is over 100 m [59], which is much longer than the side length of a typical plot. In a plot of regular size, the line-of-sight (LOS) paths between nodes may be obstructed, resulting in None-Line-of-Sight (NLOS) situations, in particular when the plot is located within a natural, dense stand. Even the UWB technology offers the great potential of achieving high ranging accuracy through its ability to resolve multipath and penetrate obstacles in harsh environments [60], its localization performance may be reduced by NLOS propagation. Although UWB signal cannot penetrate metal media or thick wall, it does have capability of penetrating trees or weed, which was supported by measuring some of the plots in this study where NLOS were true. Furthermore, several techniques have been proposed to reduce the impact of NLOS range estimates on the estimated agent position [60]. Conventionally, the RSSI is not popularly used for UWB because it does not exploit fine space resolution of impulsive signals [61]. In our method, at least three points are required in order to calculate the position on the plane, which fitted well for RSSI algorithm and four UWB Anchors. In every calculation, we need to deal with four positions (Q_1_ (X_1_, Y_1_), Q_2_ (X_2_, Y_2_), Q_3_ (X_3_, Y_3_), and Q_4_ (X_4_, Y_4_)) obtained from four tri-UWB Anchors combinations. If one position has a stronger signal, it will be given more weight (see Equation (10)). Overall, extension of the results to typical large plots, in particular when they are located in dense stands, should be taken with caution and further studies on this topic should be carried out to confirm our findings. Alternatively, measurements can be done based on four sensors for every 10 m × 10 m area. This seems feasible, at least economically, since each UWB sensor costs less than twenty dollars. 

The SLAM algorithm, convex hull algorithm, and point cloud algorithm used in other studies [4,25,27,37] usually need mobile phones or computers with 32- or 64-bit capacity. The device reported in this paper only needs the STC15, an 8-bit microprocessor, with the price being less than one dollar. In terms of computation, our method only needs four operations to obtain the estimates of DBH and tree position, making it much simpler than the other algorithm-based methods. 

## 6. Conclusions

The novelty of the dendrometer reported here is that the device can measure tree identification, position, and DBH concurrently. Specifically, we integrated several advanced technologies (UWB, QR code, altitude sensor, and so on) into the device to improve the measurement accuracy. The experimental results show that this device can be used to accurately estimate DBH and tree positions. Additionally, the device is cheap and easy to use. Nonetheless, we will continue to improve the device, in particular, improving the measurement accuracy of DBH for large trees (i.e., with DBH > 50 cm) and tree positions in complex environments. 

## Figures and Tables

**Table 1:** Descriptive statistics of the device’s components ^1^.

Component	Chip Model /Type	Interface Type	Parameter	Function
Microprocessor	STC15W4K56	SPI, I2C, Digital, Serial port, etc.	SRAM: 4 KB; Flash: 56 KB;	Data processing
QR scanner	M800	Serial port	Resolution: 20 mil;	QR scanning
Analog-to-digital sampling module	ADS1115	I2C, Analog	16 bits; 4 channels	AD sampling
UWB module	D-DWM-PG1.7	Serial port	Resolution: 1 cm; Range: 0–50 m	Distance Measurement
Altitude sensor	JY901B	Serial port	Resolution: 1 cm	Altitude Measurement
Bluetooth	HC-06	Serial port	Range: 0–15 m	COMM with upper computer
SD card	microSD	SPI	2 GB	Data storage
Angle sensor	P3014-V1	Analog	Resolution: 0.088°	Angle Measurement
Display	OLED	SPI	128 × 64 pixels	Data display
Keyboard	PVC	Digital	7 keys	Command input
Power management circuit	TP4056, DW01, AMS1117, etc.	Digital, Power	Input: 3.7–4.2 V, 5 V; Output: 3.3 V, 5 V	Power management
Battery	Lithium battery	Power	4000 mAh	Power supply

^1.^ SPI, serial peripheral interface; I2C, inter-integrated circuit; KB, kilobyte; GB, gigabyte; SRAM, static random-access memory; COMM, communication; V, voltage; mAh, milliampere-hour.

**Table 2:** Descriptive statistics of the sample plots ^1^.

Plot	Number of Trees	Dominant Species	Slope (°)	DBH (mm)			
				Mean	Max	Min	Std
1	16	S1, S2, S3	3.1	140.31	280.32	59.29	61.29
2	19	S1, S4	5.5	136.33	183.91	83.34	28.74
3	15	S1, S5	6.8	144.82	210.24	86.07	32.57
4	18	S2, S3, S6	15.3	153.72	334.63	70.54	75.13
5	28	S1, S3, S6	28.7	125.90	215.37	67.14	49.19
6	37	S7	4.8	102.43	153.97	52.75	30.38
7	30	S7	5.9	112.91	219.90	51.19	40.16
8	24	S2, S3, S7	18.3	179.74	340.21	52.60	88.00
9	20	S3, S7, S8	26.0	118.09	187.99	53.94	39.40
10	18	S7, S9	33.2	127.31	209.57	67.67	45.60

^1.^ Std, standard deviation; S1, Sapindus mukurossi Gaertn; S2, Cinnamomum camphora; S3, Magnolia denudata; S4, Michelia maudiae Dunn; S5, Ginkgo biloba; S6, Liriodendron chinensis; S7, Cunninghamia lanceolata; S8, Magnolia Grandiflora; S9, Camellia japonica.

**Table 3:** Accuracy of DBHs measured by the device based on a comparison with the reference DBHs measured by a caliper.

Plot	BIAS (mm)	relBIAS (%)	RMSE (mm)	relRMSE (%)
1	−2.04	−2.04	6.65	5.76
2	−1.22	−0.64	3.52	2.29
3	5.13	3.50	6.44	4.43
4	3.34	3.54	6.84	5.27
5	1.87	1.85	5.94	5.01
6	2.40	2.65	3.48	3.85
7	2.33	2.53	4.79	4.11
8	3.33	3.17	5.93	4.96
9	3.25	2.41	4.72	4.77
10	−0.62	−0.25	6.42	4.76
Total	1.89	1.88	5.38	4.53

**Table 4:** Accuracy of tree positions in the x- and y-axis directions estimated by the device.

Plot	X (cm)		Y (cm)		ρ_xy_
	BIAS	RMSE	BIAS	RMSE	
1	6.51	20.21	−6.40	19.72	−0.22
2	−3.97	18.45	−12.07	21.73	−0.26
3	13.65	16.82	3.67	18.44	0.13
4	0.26	23.54	−3.86	23.06	−0.21
5	14.88	26.59	−3.75	25.17	−0.12
6	−8.19	18.05	10.58	27.94	−0.09
7	−8.55	19.37	3.19	25.12	−0.11
8	3.36	23.93	−9.57	24.03	0.04
9	−1.89	12.94	24.69	28.43	0.23
10	−5.50	33.96	−9.63	24.71	0.30
Total	−0.80	21.91	1.21	24.62	−0.07

**Table 5:** Summary statistics of the error of the distance (Ed) between the estimated point and the reference point.

Plot	Ed (cm)			
	Mean	Max	Min	Std
1	26.21	47.10	11.20	10.84
2	26.19	66.18	12.56	11.25
3	23.44	47.84	11.58	8.88
4	30.84	58.23	10.85	11.61
5	33.98	69.34	12.18	13.26
6	29.42	68.47	6.18	15.53
7	29.37	60.12	4.78	11.96
8	31.99	58.87	10.05	10.77
9	28.00	64.92	3.84	13.36
10	38.08	76.40	11.27	16.99
Total	38.06	76.40	3.84	13.53
